# Transposition of *Mboumar-9*: Identification of a New Naturally Active *mariner*-Family Transposon

**DOI:** 10.1016/j.jmb.2008.07.044

**Published:** 2008-10-10

**Authors:** Martín Muñoz-López, Azeem Siddique, Julien Bischerour, Pedro Lorite, Ronald Chalmers, Teresa Palomeque

**Affiliations:** 1 Departamento de Biología Experimental, Área de Genética, Universidad de Jaén, 23071 Jaén, Spain; 2 Department of Biochemistry, University of Oxford, South Parks Road, Oxford OX1 3QU, UK; 3 University of Nottingham, School of Biomedical Sciences, Queens Medical Centre, Nottingham NG7 2UH, UK

**Keywords:** TIR, terminal inverted repeat, stDNA, satellite DNA, ORF, open reading frame, MITE, miniature inverted-repeat transposable element, MBP, maltose-binding protein, *mariner*, transposon, satellite DNA, MITE, DNA recombination

## Abstract

Although *mariner* transposons are widespread in animal genomes, the vast majority harbor multiple inactivating mutations and only two naturally occurring elements are known to be active. Previously, we discovered a *mariner*-family transposon, *Mboumar*, in the satellite DNA of the ant *Messor bouvieri*. Several copies of the transposon contain a full-length open reading frame, including *Mboumar-9*, which has 64% nucleotide identity to *Mos1* of *Drosophila mauritiana.* To determine whether *Mboumar* is currently active, we expressed and purified the *Mboumar-9* transposase and demonstrate that it is able to catalyze the movement of a transposon from one plasmid to another in a genetic *in vitro* hop assay. The efficiency is comparable to that of the well-characterized *mariner* transposon *Mos1*. Transposon insertions were precise and were flanked by TA duplications, a hallmark of *mariner* transposition. *Mboumar* has been proposed to have a role in the evolution and maintenance of satellite DNA in *M. bouvieri* and its activity provides a means to examine the involvement of the transposon in the genome dynamics of this organism.

Transposable elements catalyze the movement of DNA from one genomic location to another. They are powerful forces of genetic change and have played a significant role in the evolution of many genomes. RNA transposons (class I) function via reverse transcription of an RNA intermediate, while DNA transposons (class II) generally move by a cut-and-paste mechanism in which the transposon is excised from one location and reintegrated elsewhere.

The *mariner* family of DNA transposons is probably the most widely distributed family of transposable elements in nature, represented in such diverse taxa as fungi, ciliates, rotifers, insects, nematodes, plants, fish and mammals.^1–3^ Members of the family share three characteristics: a transposase with a DDD catalytic motif, short terminal inverted repeats (TIRs), and a TA dinucleotide target site that is duplicated upon insertion. During transposition, the two ends of the transposon are brought together by oligomerization of the bound transposase to generate a synaptic complex. The transposase then cleaves the DNA at each transposon end and promotes integration of the excised transposon at a new target site.^4–8^

Satellite DNA (stDNA) consists of long arrays of tandemly repeated sequences located in genetically silent heterochromatic regions.^9^ Analysis of the stDNA of the ant, *Messor bouvieri*, revealed the presence of several copies of a *mariner* transposon termed *Mboumar*.^10^ The transposon is about 1287 bp in length, is flanked by 32-bp inverted repeats, and contains an open reading frame (ORF) coding for a transposase of 345 amino acids with up to 68% amino acid identity to the *Drosophila mauritiana Mos1* transposase. Like other *mariner* transposases, the transposase has two domains: an amino-terminal region containing a helix–turn–helix motif necessary for the recognition and binding of TIRs, and a carboxy-terminal catalytic domain harboring a DD34D catalytic motif.

Miniature inverted-repeat transposable elements (MITEs) are short (80–500 bp) transposon-like elements present in large numbers in many eukaryotes, particularly plant species,^11,12^ and occasionally in bacteria.^13,14^ Although they have TIRs and are flanked by target site duplications, they generally lack transposase coding potential and are therefore presumably dependent on full-length autonomous transposons for mobility. Multiple copies of a 130-bp MITE-like element, termed IRE-130, have been detected in the stDNA of *M. bouvieri*.^10^ Five copies of the element were found to harbor copies of the *Mboumar* transposon inserted at the same TA dinucleotide.^10^ This suggested that stDNA and IRE-130 could represent hot spots for *Mboumar* insertions.

We have expressed and purified the *Mboumar* transposase from a naturally occurring copy of the transposon in *M. bouvieri*. We demonstrate here that the transposase is fully active and can perform transposition *in vitro*. Despite the wide phylogenetic distribution of *mariner* elements in nature, almost all harbor inactivating point mutations or deletions. Consequently, *Mboumar* is only the third naturally occurring, active, classical (DD34D) *mariner* transposase discovered to date (after *Mos1*^15,16^ and *Famar1* from the European earwig, *Forficula auricularia*^17^). The widespread presence of the *Mboumar* transposon in *M. bouvieri* stDNA suggests a positive role for the transposon in the development and/or maintenance of the DNA. Transposition has been hypothesized to play a role in the evolution of stDNA^18^ and the activity of the *Mboumar* transposase could provide us with the means to examine this idea.

## *In vitro* excision of the *Mboumar* transposon by *Mboumar-9* transposase

**Fig. 1:**
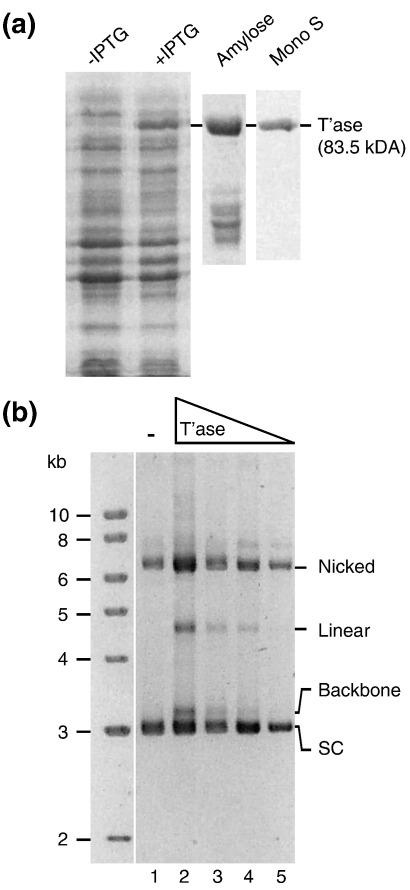
Purification and excision activity of the *Mboumar-9* transposase. (a) SDS-PAGE electrophoresis of fractions from various steps of the *Mboumar-9* transposase purification procedure. An MBP–Mboumar-9 transposase fusion protein was expressed in *E. coli* Rosetta 2 (Novagen) using the pMAL-c2X expression system from New England BioLabs. Expression and purification were performed essentially as described in the supplied instruction manual. Briefly, cells were lysed by French press and centrifuged, and the soluble fraction was passed over amylose resin. MBP-transposase was eluted with maltose and then purified further by cation-exchange chromatography on a MonoS HR5.5 column (Amersham Pharmacia). Elution was with a 20 column volume gradient from 0.05 to 1 M NaCl in Hepes buffer. Lane 1, uninduced cleared cell lysate of *E. coli* Rosetta 2 cells harboring the MBP-transposase expression plasmid (pRC675); lane 2, cleared cell lysate from the same culture an hour after induction with IPTG; lane 3, eluate from the amylose column; lane 4, purified protein after cation-exchange chromatography. The MBP transposase fusion protein is 83.5 kDa. (b) *In vitro* cleavage assay. DNA cleavage was performed at 28 °C for 5 h in a total volume of 30 μl. The reaction contained 9 nM of the transposon donor plasmid pMboumar-9, which carries a complete wild-type copy of *Mboumar-9*. The standard reaction buffer was 25 mM Hepes (pH 7.9) supplemented with 12.5 mg/ml bovine serum albumin, 2 mM DTT, 100 mM NaCl, 10% glycerol and 10 mM MgCl_2_ or MnCl_2_. Lane 1, no transposase; lanes 2, 3, 4 and 5, reactions with 27, 9, 3 and 1 nM transposase, respectively.

Several *Mboumar* transposons in *M. bouvieri* contain a full-length (complete) transposase ORF. We chose to study the ORF from the *Mboumar-9* copy of the transposon because it has preserved all of the critical sequence motifs previously identified in other *mariner* transposases (the complete nucleotide sequence of the element was published by Palomeque *et al*.^10^). These include the DNA binding helix–turn–helix motif, the bipartite nuclear localization signal, the WVPHEL linker motif and the DD34D catalytic triad. The *Mboumar-9* transposase was fused to the maltose-binding protein (MBP) affinity-purification tag by inserting the ORF into the pMAL-c2X plasmid. The MBP–Mboumar-9 fusion was expressed in *Escherichia coli* and purified as described (Fig. 1a).

To test whether the *Mboumar-9* transposase was capable of transposon excision, a plasmid harboring a copy of *Mboumar-9* (pMboumar-9) was incubated with purified transposase and the reaction products were examined by agarose gel electrophoresis (Fig. 1b). Excision of the transposon is expected to release a 3155-bp fragment corresponding to the plasmid backbone. Such a fragment is observed in the presence, but not the absence, of transposase (Fig. 1b). This result demonstrates that the *Mboumar-9* transposase is proficient for the excision step of the reaction. We did not detect a fragment corresponding in size to the excised transposon, suggesting that it reacted further to produce integration products. Among the cut-and-paste transposons, the excised linear transposon is a transient species not usually observed at late time points such as these because it rapidly undergoes inter- and/or intramolecular insertions (e.g., Refs. 19–21).

## Integration of *Mboumar-9*

**Fig. 2:**
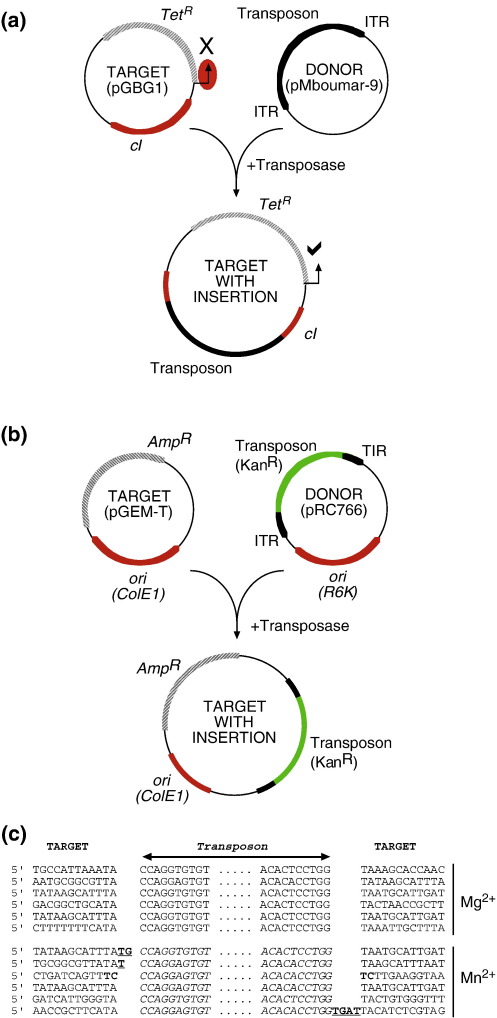
Genetic assays for transposition. (a and b) Schematic representations of the two *in vitro* hop assays for transposition. (a) Transposition of *Mboumar-9* from pMboumar-9 to a target plasmid, pGBG1. The *cI* gene on pGBG1^22^ acts as a trap for unmarked mobile genetic elements, as described in the text. (b) Transposition of a mini-*Mboumar-9* transposon in which the TIRs flank a gene for kanamycin resistance (plasmid pRC766), as described in the text. Drug resistance markers are as follows: *Tet*^*R*^, tetracycline resistance; *Kan*^*R*^, kanamycin resistance; *Amp*^*R*^, ampicillin resistance. *ori*, plasmid origin of replication; *cI*, lambda phage *cI* repressor gene. (c) DNA sequences of transposon integration sites in plasmid pGBG1. *In vitro* transposition reactions were incubated for 10 h at 28 °C in a 30-μl reaction volume containing 9 nM of each of the donor and target plasmids and 10 nM purified transposase in the standard reaction buffer defined in Fig. 1. Five microliters was used to transform *E. coli* DH5α cells. Plasmid DNA from the colonies obtained on selective medium was examined by restriction analysis for potential transposition products. DNA sequencing to confirm the transposon junctions was initiated from primer sites flanking the ends of the *cI* repressor gene. The underlined bases in the figure are extra (non-target) nucleotides at the insertion junctions. The mechanism by which these nucleotides were added is unclear.

We tested the *Mboumar-9* transposase for transposition activity in a genetic *in vitro* “hop” experiment. In this assay, purified transposase is incubated with a transposon donor and a target plasmid. Transposition events, from donor to target, are recovered by genetic transformation of bacteria and selection of the appropriate antibiotic resistance markers (Fig. 2a and b).

We implemented two variations of this assay. The first version employed a “transposon trap” strategy (Fig. 2a). In this assay, the target plasmid (pGBG1) harbors the *tetA* (tetracycline resistance) gene under the control of the bacteriophage lambda *pR* promoter. The promoter is repressed by the product of the adjacent lambda *cI* repressor gene, rendering the host cell sensitive to the antibiotic. A mutation, such as a transposon insertion, in the *cI* gene will inactivate the repressor, rendering the host cell resistant to tetracycline. *In vitro* transposition was performed with pMboumar-9 as the donor and the pGBG1 trap as target. The reactions were first evaluated by agarose gel electrophoresis. As expected, a 3155-bp fragment corresponding to the plasmid backbone was detected, indicating that excision of the transposon had occurred (data not shown). The reaction also revealed a set of high molecular weight bands that may correspond to transposon integration products.

To assay for transposon integration into the target plasmid, the reaction mixture was used to transform *Escherichia coli*. Colonies were obtained only from complete transposition reaction mixtures. No colonies were obtained when the donor, target or transposase were omitted from the mixture. DNA was recovered from 27 colonies and examined by restriction analysis. All 27 plasmids had an insertion within the *cI-tetA* cassette of the size expected for *Mboumar-9*. The transposon–target junctions in six of these plasmids were determined by DNA sequencing. In all six cases, the junctions were precise and insertions had occurred into TA dinucleotide target sites in the *cI* gene (Fig. 2c). Transposition was also performed with Mn^2+^ instead of Mg^2+^ as the catalytic metal ion. In this condition, some of the transposon ends were imprecise and a few of the insertions occurred at target sites other than TA (Fig. 2c, Mn^2+^ panel).

The *cI* region of the trap plasmid represents a very limited target region for transposition. To better estimate the frequency of transposition, we used a second version of the *in vitro* hop assay that was designed to detect integration events in non-essential regions of a target plasmid (Fig. 2b). This strategy could also be used to identify potential hot-spot sequences in the target. We constructed a mini-*Mboumar-9* donor plasmid in which the gene for kanamycin resistance is flanked by the TIRs of the transposon (pRC766). The relative frequency of transposition was measured by counting the number of colonies obtained after selection with kanamycin and ampicillin (resistance to the latter antibiotic is coded for by a gene on the target plasmid). To exclude the confounding effect of double transformation by both donor and target, the donor plasmid was based on a conditional R6K origin of replication, which is unable to function in the *pir*^*−*^ recipient strain. Similar to what was observed in the previous experiment, colonies were obtained only from complete reaction mixtures, and not when the transposase, donor or target were omitted from the reaction. Using this assay, we calculated the relative transposition efficiency (the proportion of target molecules carrying transposon insertions) to be 10^− 3^ in the presence of Mg^2+^, and 10^− 4^ when Mn^2+^ was substituted for Mg^2+^.

Taken together, these assays demonstrate that *Mboumar-9* has easily detectable transposition activity *in vitro*. Since the reactions were performed in physiological Mg^2+^, pH and salt conditions, it seems likely that *Mboumar-9* is active in its natural host *M. bouvieri*.

## Target specificity of *Mboumar-9*

A previous study indicated that *Mboumar* is highly represented in the stDNA of *M. bouvieri*.^10^ Any number of different factors, such as a preference for heterochromatin, could potentially give rise to an insertional bias *in vivo*.^23^ However, other transposons have insertion hot spots *in vivo* or *in vitro* that are probably determined by the structure of naked DNA.^19,21,24–26^ DNA structure is determined directly by nucleotide sequence and is dictated by factors such as AT/GC content and the phasing of repetitive regions.^27^

To discover whether *Mboumar-9* has enhanced affinity for *M. bouvieri* stDNA, we performed an *in vitro* hop assay using stDNA as a target. The target plasmid (pMBSAT) carries a 575-bp fragment consisting of seven tandem repeats of the 79-bp stDNA monomer. Transposition products were analyzed by their NotI restriction digestion pattern (not shown). Nine of the 45 transposition clones tested contained insertions within the stDNA fragment. The proportion of insertions within stDNA (0.20) is not statistically different from the ratio expected if insertions were random. This is true whether the theoretical ratio is based on the relative lengths of non-essential regions on the plasmid (0.19) (χ^2^ ≤ χ_0.95_^2^) or on the relative numbers of available TA dinucleotide target sites (0.26) (χ^2^ ≤ χ_0.95_^2^). As a control, transposition was also performed with the parental plasmid pGEM-T. Transposition efficiency was evaluated by measuring the proportion of total insertions in the *lacZ* gene. The proportion of insertions in *lacZ* was no different from the ratio expected if insertions were random (data not shown). Thus, the target plasmid does not contain any significant hot or cold spots that would influence insertion frequencies.

The IRE-130 MITE is 130 bp in length and contains only four TA dinucleotides that are potential target sites for *Mboumar* integration. Five of the *Mboumar* copies identified in *M. bouvieri* stDNA were inserted at the same TA dinucleotide in different copies of IRE-130.^10^ To determine whether this TA dinucleotide represents a hot spot for *Mboumar* insertion, an *in vitro* hop experiment was performed using a target plasmid (pIRE130) harboring a single copy of IRE-130. We examined 53 independent insertions, but only 3 were present within IRE-130. The remaining insertions were elsewhere in the plasmid. Only one of the IRE-130 insertions was at the TA dinucleotide favored *in vivo*. As in the previous experiment, the proportion of insertions in IRE-130 (0.06) was no different from that expected if insertions were random (0.05 and 0.04, theoretical ratios based on the lengths of non-essential regions on the plasmid, and on the numbers of TA dinucleotide target sites, respectively [χ^2^ ≤ χ_0.95_^2^]). These results suggest that Mboumar is not strongly biased towards the intrinsic, sequence-dictated structure of *M. bouvieri* stDNA or the IRE-130 MITE. Any factors responsible for targeting these sequences *in vivo* must therefore reside at a higher level of chromosome organization.

## Implications for satellite DNA evolution

*Mariner* transposons are arguably one of the most successful transposon families in existence, as evidenced by their widespread distribution in diverse eukaryotic taxa. Yet prior to this report, experimental evidence for transposition activity had only been reported for two naturally occurring classical (DD34D) *mariner* transposons: *Mos1* from *D. mauritiana*,^15,16^ and *Famar1* from the European earwig, *F. auricularia*.^17^ In contrast, the well-characterized active *mariner* transposon, *Himar1*, represents the consensus sequence derived from numerous inactive elements in the horn fly, *Hematobia irritans*.^4^ The human *Hsmar1 mariner* transposon, notably incorporated into the *SETMAR* gene by a domestication event, has also been similarly resurrected using a reconstructed ancestral sequence deduced from inactive copies.^28–30^

We have shown that *Mboumar-9* is capable of transposition *in vitro*. Transposon insertions are precise and occur at TA dinucleotide sites, which are duplicated upon insertion. Consistent with data from other *mariners*, transposition of *Mboumar* requires no proteins or cofactors other than Mg^2+^ and the transposase itself. The efficiency of transposition for *Mboumar* in our genetic *in vitro* hop assay (in the presence of Mg^2+^) is comparable to that obtained for *Mos1* and *Himar1* under similar experimental conditions,^25,31^ suggesting that their activities may be comparable. Transposition was also examined with the non-physiological ion, Mn^2+^, instead of Mg^2+^. Although transposition is often enhanced with Mn^2+^,^28,32^ transposition of *Mboumar* was reduced 10-fold in the presence of an equal concentration of this cofactor. This has also been observed with *Mos1*.^31^ Mn^2+^ also caused a relaxation in target site specificity. This has been seen for many transposition systems and is explained by evidence indicating that Mn^2+^ permits more flexible DNA strand positioning in the active site than does Mg^2+^.^28,32^

Transposition has been postulated to play an important role in the evolution of stDNA.^18^ The recombinogenic nature of transposons, their ability to proliferate and to transpose to locally restricted target sites are properties well suited to a role in the expansion, diversification and homogenization of stDNA. Indeed, some stDNA families are believed to have originated from transposable elements.^33,34^ *Mboumar* has been identified in three ant species to date: *M. bouvieri*, *Messor structor* and *Messor barbarus*.^10^ In all three species, multiple copies are localized to stDNA. Unusually for *mariners*, the vast majority of which are inactivated by multiple mutations, several copies of *Mboumar* contain intact ORFs.^10^ This suggested that *Mboumar* activity may have recently contributed to, and may continue to act on, the stDNA structure in these ant species.

The association of *Mboumar* with stDNA and the IRE-130 MITE suggested that they might represent hot spots for insertion. However, our present results suggest that Mboumar has no intrinsic affinity for these DNA sequences, although its affinity for DNA may be quite different *in vivo*. One alternative explanation is that *Mboumar* insertions favor stDNA because insertions are excluded from euchromatin either by counterselection or by the physical properties of the different genomic regions, as suggested for other transposons.^33,35^ There is also the possibility that *Mboumar* activity in stDNA provides a benefit of some kind. Current experiments directed towards understanding *Mboumar* function *in vivo* may provide an answer to these questions.

Previous analysis of *Mboumar* insertion sites in stDNA has provided at least one example where co-transposition of adjacent *Mboumar* copies could have led to mobilization of the intervening stDNA.^10^ The excision and integration activities demonstrated here for *Mboumar* provide the tools to address this prediction and unearth the role of transposition, if any, in the evolution of stDNA. It might also provide us with evidence of ongoing transposition activity that continues to shape the *M. bouvieri* genome.
